# Power-Law Distributions of Dynamic Cascade Failures in Power-Grid Models

**DOI:** 10.3390/e22060666

**Published:** 2020-06-16

**Authors:** Géza Ódor, Bálint Hartmann

**Affiliations:** Centre for Energy Research, P. O. Box 49, H-1525 Budapest, Hungary; hartmann.balint@energia.mta.hu

**Keywords:** power-grid, Kuramoto, dynamic simulation, failure cascade

## Abstract

Power-law distributed cascade failures are well known in power-grid systems. Understanding this phenomena has been done by various DC threshold models, self-tuned at their critical point. Here, we attempt to describe it using an AC threshold model, with a second-order Kuramoto type equation of motion of the power-flow. We have focused on the exploration of network heterogeneity effects, starting from homogeneous two-dimensional (2D) square lattices to the US power-grid, possessing identical nodes and links, to a realistic electric power-grid obtained from the Hungarian electrical database. The last one exhibits node dependent parameters, topologically marginally on the verge of robust networks. We show that too weak quenched heterogeneity, coming solely from the probabilistic self-frequencies of nodes (2D square lattice), is not sufficient for finding power-law distributed cascades. On the other hand, too strong heterogeneity destroys the synchronization of the system. We found agreement with the empirically observed power-law failure size distributions on the US grid, as well as on the Hungarian networks near the synchronization transition point. We have also investigated the consequence of replacing the usual Gaussian self-frequencies to exponential distributed ones, describing renewable energy sources. We found a drop in the steady state synchronization averages, but the cascade size distribution, both for the US and Hungarian systems, remained insensitive and have kept the universal tails, being characterized by the exponent τ≃1.8. We have also investigated the effect of an instantaneous feedback mechanism in case of the Hungarian power-grid.

## 1. Introduction

Modeling power grids has become a hot topic in statistical physics as electric energy infrastructure is bound to undergo huge changes in both the generation and demand sides to make it environmentally sustainable. They are a large complex, heterogeneous dynamical system, built up from nodes of energy suppliers and consumers, interconnected by a network with hierarchical modular (HMN) structure [1,2,3]. The transition from fossil to renewable energy sources poses unprecedented challenges towards the robustness and resilience of power grids, as they introduce correlated spatio-temporal fluctuations.

Unexpected changes may cause desynchronization cascades, propagating through the whole system as an avalanche, causing blackouts of various sizes. These can lead to full system desynchronization, lasting for a long time [4]. Numerous attempts have been made for understanding and forecasting power outages from several different angles [5]. Particularly, from the point of view of statistical physics of breakdown phenomena, systemic risk of failure in power infrastructure represents a particular case of a generic phenomena: the risk of system-wide breakdown in threshold activated disordered systems.

The size distributions of the outages have been found scale-free in the US, China, Norway, and Sweden in the available long time series data [6]. They have been modeled [7] by direct current (DC) threshold models with self-organized criticality (SOC) [8], arising as the consequence of self-tuning to a critical point by the competition of power demand and network capabilities. These models are similar to those of sand piles, in which redistribution avalanches are generated, when the local level exceeds a threshold value. By analyzing the statistics of seven years (from 2002 to 2008) of European Union (EU) network failures a moderate support for scale-free behavior has been found [9]. In particular, power-laws could be fitted better in countries, with so-called robust networks [10]. The categorization of robust/fragile is based on the static network topology analysis of national power-grids [11], where networks with P(k>K)=Cexp(−k/γ) cumulative degree (*k*) distribution and γ<3/2 are called robust. Restoration time was supported particularly well by a power law (PL) model in both groups, but this behavior is in accordance with findings, where human temporal response distributions have been found to be fat tail distributed. It is well known that human behavior exhibits bursty behavior [12], which raises the question whether the observed PL-s are the consequence of the power-grid function itself or related to the bursty behavior of system maintenance procedures. One of the aims of our study is to investigate whether such PL-s can be reproduced by more realistic power-grid models than the first attempts made using simple threshold ones.

The framework of direct current DC threshold models [7] can be extended by taking into account the real power flow in alternating current (AC) networks by modeling via the second order Kuramoto equation [13]. A number of studies exist, which focus on the synchronization and stability issues. In [14], the authors show that the coupling strength of the power grid models behaves differently, depending on the heterogeneity of the nodes; synchronization appears in highly heterogeneous complex networks, where nodes show different characteristics. Our work considers an even more heterogeneous system derived from real data and also goes beyond the bimodal Gaussian self-frequency approximations. Choi et al. [15,16] use various frameworks to test the effect of inertia on the speed of synchronization. Their results imply that large inertia induces slower synchronization. In their works, Dörfler et al. [17,18] examine synchronization and stability in power networks and other complex networks, applying non-uniform (heterogeneous) parameters to the Kuramoto-model. Using the real topology of the Italian transmission network, Fortuna et al. [19] find that the class of Kuramoto-like models with bimodal distribution (sources and consumers) of the frequencies is the most appropriate mapping between oscillators and power system nodes. The same network and modeling approach is also used in [20], concluding that the synchronization transition is hysteric for sufficiently large masses, but, for Italian high voltage power grid, the transition is largely nonstrategic, due to the low value of the average connectivity. Future spread of distributed generation is modeled in [21], while using non-uniform parameters for power system nodes. The results of the authors show that realistic (non-optimal) topologies have wider phase differences between connected nodes, which leads to less homogeneously transmitted power, but no significant differences have been observed in case of node removal. Smaller topologies are used in [22] to test the extension of the Kuramoto-model with voltage dynamics to study the voltage-angle stability of power systems. The authors of [23] introduce a method to estimate coupling strength of power grids, which is a crucial parameter of the Kuramoto-model; proper knowledge of such parameters can help in maintaining stability of the power system even in the presence of large transients. The recent work by Taher et al. [24] proposes a time-delayed feedback control to the Kuramoto-model and test it on a realistic topology, with complex bimodal self-frequency distributions.

Our study goes beyond the synchronization stability issues, by generating failure cascade distributions, which had only been considered in DC models. Solving AC power flow equations is a significant computational challenge. The DC approach limits this by linearizing the equations and it has been used in large-scale simulations. It considers active powers, but ignores reactive ones and transmission losses [25]. Its efficiency approximates the AC power flow, without being iterative and complex [26,27]. It misrepresents transmission line flows by less than 5%, but about 10 times faster than the exact solution provided by the AC load flow approach [28]. However, while, for DC threshold models, SOC critical transition is established, for AC threshold models we have no knowledge how the underlying second order Kuramoto model, which has a first order transition [3], affects the avalanche size distributions. One of the main objective of our study is to show how scale-free avalanches can occur in the AC Kuramoto threshold model as we increase the network heterogeneity.

The synchronization and stability can be deduced from the power transfer behavior of a load/supply AC electrical circuit and it turns out to be the generalization of the Kuramoto model [29] with inertia. The Kuramoto model below $d<d_{l}=4$ does not exhibit real phase transition to a synchronized state, but a smooth crossover only [30]. In real life, we can observe partially synchronized states. The second order Kuramoto equation is also expected to have $d_{l}=4$, and in lower graph dimensions the transition point shifts to infinity with the system size and hysteresis behavior emerges [3].

While most of the SOC models are homogeneous, which means that all nodes and interactions are the same and the connection matrix is regular deterministic, in real life all kinds of heterogeneity can occur in the connection network topology as well as in the node/interaction parameters. Highly heterogeneous, also called disordered with respect to the homogeneous, system can experience rare-region effects altering critical dynamics [31]. These rare regions, which are locally in another state than the whole, evolve slowly and contribute to the global order parameter, causing slow dynamics and fluctuations. They can generate so-called Griffihts Phases (GP) [32] in an extended region around the critical point, causing slowly decaying auto-correlations and burstyness [12]. In synchronization models, such rare regions can cause frustrated synchronization and chimera states [33,34,35]. These result in non-universal PL distributions of the desynchronization events below the transition point [3,36,37]. In Ref. [3], we provided numerical evidence for this by modeling a sudden drop of global coupling of the second order Kuramoto model defined on 2D square lattices and on large synthetic power-grids.

Very recently, dynamical modeling of cascade failure has been introduced combining the second order Kuramoto with power transfer thresholds [38]. The identification of critical lines of transmission in different national power grids has been determined. We follow this method to investigate the desynchronization duration distributions via measuring the number of failed lines following a node removal event. We shall compare the results obtained on 2D square lattices with those of the US high voltage power-grid and the Hungarian power-grid with 418 nodes that we generated from our network providers.

Modeling power-spectra of renewable energy sources has been done in the case of wind farms and solar cells [39]. The effects of sudden weather changes and the strong spatio-temporal correlations decrease the stability of power grids. The power output of a single unit deviates largely from the normal distribution, but this non-Gaussian behavior also remains for the aggregated power of farms. Therefore, the central limit theorem, predicting a convergence to Gaussian for independent data sets with defined standard deviation, does not apply. Here, we shall also investigate the effects of replacing Gaussian self-frequency distributions to exponential ones in the case of our power-grid models. In particular, we test the robustness of the scale-free behavior of outage distributions, by the replacement of all nodes to non-Gaussian.

## 2. Models and Methods

The main purpose of using the Kuramoto-model is to examine the cascade failures. Transmission System Operators traditionally use a static approach for such analysis, which means that they start the simulation at a fixed operating point by performing a load-flow, trip the faulty line (remove the edge from the graph), and then perform another load-flow at this different operating point. While this method is simple, it fails to capture the dynamic response of the units (generators and loads) in the system, since the iterative nature of load-flow calculations aims to create a numerical solution; if necessary, by linearization and simplification. In the contrary, the Kuramoto-model starts the simulation at a fixed operating point by performing thermalization (which is a dynamic process), tripping the faulty line, and examining the unfolding transient, which will reveal dynamic response of the units.

The evolution of synchronization is based on the swing equations [40] set up for mechanical elements with inertia by the second order Kuramoto equation [13]. For a network of *N* oscillators with phase $\theta_{i}\left(t\right)$:

(1) $$\begin{array}{lll} \dot{\theta_{i}}\left(t\right) & = & \omega_{i}\left(t\right)\\ \dot{\omega_{i}}\left(t\right) & = & \omega_{i}^{0}-\alpha \dot{\theta_{i}}\left(t\right)+K\sum_{j=1}^{N}A_{ij}\sin\left[\theta_{j}\left(t\right)-\theta_{i}\left(t\right)\right]\;, \end{array}$$

where α is the damping parameter, describing the power dissipation, *K* is the global coupling, being related to the maximum transmitted power between nodes and $A_{ij}$, which is the weighted adjacency matrix of the network, containing admittance elements. Very recently, this equation has been refined with the aim of application for the German HV power-grid by [24]

(2) $${\ddot{\theta }}_{i}+\alpha \;{\dot{\theta }}_{i}=\frac{P_{i}}{I_{i}\;\omega_{G}}+\frac{K_{i}}{I_{i}\;\omega_{G}}\;\sum_{j=1}^{N}A_{\mathrm{ij}}\;\sin\left(\theta_{j}-\theta_{i}\right)\;.$$

Generator units ($P_{i}>0$) and loads ($P_{i}<0$) are modeled with a bi-modal probability distribution with peaks at mean values of power sources and sink. The authors assume homogeneous transmission capacities, thus $K_{i}=K$. The dissipation parameter 0.1[1/s]≤α≤1[1/s] and moments of inertia at the nodes is also considered to be homogeneous: $I_{i}=I=40\;\times \;10^{3}\;\left[{\mathrm{kgm}}^{2}\right]$, which approximately equals the moment of inertia of a 400 MW power plant. The adjacency matrix is constructed of binary elements, 1 represents connection and 0 represents the lack of it. The authors cite that previous applications of the Kuramoto equation had a significant limitation, as all generators and loads were handled with a bi-modal δ-distribution, where all of the units had the same power. However, the proposed method uses empirical data for $P_{i}$ only and all other parameters are handled in a uniform way. In the following, we extend this, as follows.

When considering Equation (2), the following statements can be made:

1. α dissipation factor is chosen to be equal to 0.4/[1/s], which value will be used in this paper as well
2. in real power systems, the *i*-th node has connection both to generators and loads, thus $P_{i}$ parameter of the equation can be written as

(3) $$P_{i}=P_{\mathrm{Gi}}-P_{\mathrm{Li}}\;,$$

where $P_{G}$ represents generators (production), $P_{L}$ represents loads (consumption).

For a given node, the ratio of $P_{Gi}$ and $P_{Li}$ shows significant dependence on the voltage of the node and the size of the supplied service area. If the node serves as the connection point of a power plant, $P_{Li}\;≅\;0$, since only self-consumption of the plants has to be considered as a load. If the node only supplied consumers, $P_{Gi}=0$. It has to be noted that due to the increasing number of distributed generators, such purely consuming nodes are becoming less frequent. The third case is the most typical, when the node connects both supplies and loads. In such cases, the ratio of $P_{\mathrm{Gi}}$ and $P_{\mathrm{Li}}$ will determine not only that a certain node will behave as a net producer or a net consumer, but also the moment of inertia for that service area. Exact ratios might also depend on the actual load state, season, day of the week, etc., where variations could be addressed by using so-called characteristic load states (e.g., summer and winter peaks).

The $I_{i}$ moment of inertia can be considered as a sum of two contributions: inertia of generators and inertia of loads. In large power systems, the cumulative moment of inertia of power plants exceeds that of the loads by magnitudes, so load inertia is often neglected. However, in the examined network model, there are numerous subsystems, where the power (and thus the inertia) of generators is very low or even zero. The relation between the body moment of inertia, apparent power $S_{i}$ and *H* inertia constant is:

(4) $$I_{i}=\frac{2\;H\;S_{i}}{{\omega_{G}}^{2}}$$

The magnitude of the inertia constant is highly dependent on the type of the power plant (see Table 1) and the load mix (see Table 2) as well, thus uniform handling of $I_{i}$ is a simplification of modeling.

The $K_{i}$ couplings represent the amount of power that can be transmitted from the *i*-th node. If elements of $A_{\mathrm{ij}}$ adjacency matrix take up binary (0/1) values, the dimension of the coupling is power: $\left[K_{i}\right]=\mathrm{MW}$. Such power values are usually available in the database of system operators as operation limits. These operational limits can be based on thermal limits (to avoid overloading of the conductor) or limited capabilities of the infrastructure (measurement transformers, switch gear, etc.). The operational limits show large dependence on voltage level, age of the infrastructure, and seasons, thus uniform handling of this parameter is also a simplification of modeling. In conclusion, returning to the equation by [24] for $P_{i}$, $K_{i}$ and $I_{i}$ empirical distribution values can be used instead of an uniform characterization.

Taking into consideration that multiple generators and loads can be connected to the same node, cumulative values (e.g., net load) will be marked by area,i index instead of the *i* index. Transforming Equation (2), $P_{\mathrm{area},i}$ will represent the net load of a certain area:

(5) $${\ddot{\theta }}_{\mathrm{area},i}+\alpha \;{\dot{\theta }}_{\mathrm{area},i}=\frac{P_{\mathrm{area},\mathrm{Gi}}-P_{\mathrm{area},\mathrm{Li}}}{I_{\mathrm{area},i}\;\omega_{G}}+\frac{K_{\mathrm{area},i}}{I_{\mathrm{area},i}\;\omega_{G}}\;\sum_{j=1}^{N}A_{\mathrm{ij}}\;\sin\left(\theta_{j}-\theta_{i}\right)$$

Using the relation (4), we are able to express the inertia constant of the service area:

(6) $$I_{\mathrm{area},i}=\frac{2\;H_{\mathrm{area}}\;S_{\mathrm{area},i}}{{\omega_{G}}^{2}}\;,$$

If the area only consists of generators, $H_{\mathrm{area}}=H_{\mathrm{Gi}}$ and the value can be determined based on the composition of the power plant portfolio, while using Table 1. For European power systems, these values are expected to be between 6 s and 1 s in 2030, depending on the power plant portfolio [42]. If the area consists of both generators and loads, the value of $I_{\mathrm{area},i}$ can be calculated taking into consideration the inertial response of both generators and loads

(7) $$I_{\mathrm{area},i}=\frac{\sum \left(2\;H_{\mathrm{Gi}}\;S_{\mathrm{Gi}}\right)}{{\omega_{G}}^{2}}+\frac{\sum \left(2\;H_{\mathrm{Li}}\;S_{\mathrm{Li}}\right)}{{\omega_{G}}^{2}}\;,$$

where $S_{\mathrm{Gi}}$ is the power of single generator units, $S_{\mathrm{Li}}$ is the power of single load units. Value of $H_{\mathrm{Gi}}$ can be chosen from Table 1, while in case of $H_{\mathrm{Li}}$ certain empirical values can be used (see Table 2). In this paper, it is assumed that 60–70% of total load is of rotating machines (H=0.5 [s]), and the remaining 30–40% load is of low inertia units (H=0.1 [s]), $H_{\mathrm{Li}}$ equals:

(8) $$\begin{array}{lll} H_{\mathrm{Li}} & = & \frac{\sum \left(0.5\;s\;S_{\mathrm{Li}}\;\left[0.6\cdots 0.7\right]+0.1\;s\;S_{\mathrm{Li}}\;\left[0.4\cdots 0.3\right]\right)}{S_{\mathrm{Li}}} \end{array}$$

(9) $$\begin{array}{lll} & = & \sum 0.5\;s\;\left[0.6\cdots 0.7\right]+0.1\;s\;\left[0.4\cdots 0.3\right]=\left[0.34\cdots 0.38\right]\;s\approx \;0.36\;s \end{array}$$

**Figure 1:**
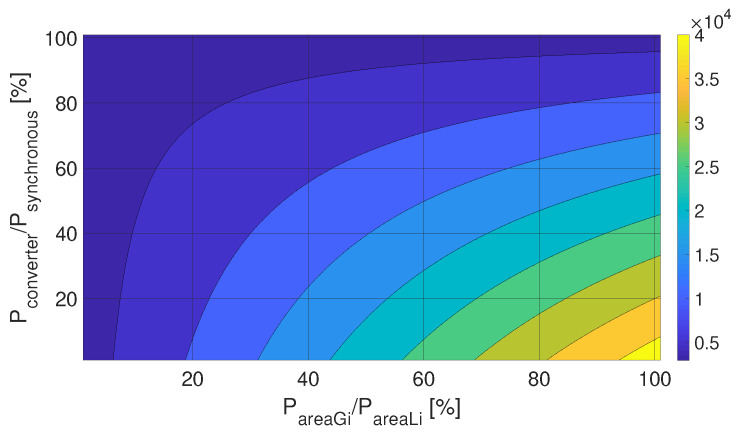
Moment of inertia for a 400 MW node, depending on the proportion of locally generated power and the share of converter-based generation units.

An illustrative example is shown to underline the importance of properly assessing $I_{\mathrm{area},i}$. In the paper by [24], $I_{i}=I=40\times 10^{3}\;\left[{\mathrm{kgm}}^{2}\right]$ was used as a representation of a 400 MW power plant, which by substituting into Equation (6) will result $H_{\mathrm{area}}=4.93$ [s]; this will be used as $H_{\mathrm{Gi}}$ in the following example. Figure 1 shows how the moment of inertia varies for a 400 MW node, depending on the proportion of locally generated power and the share of converter-based generation units, which have no inertia. The values on the figure vary between 2918 and $42879\;[{\mathrm{kgm}}^{2}]$, which emphasizes the importance of using different inertia values for the nodes in such models. E.g., in the case of the Hungarian model, only 10% of the nodes can be represented as purely generation ones and the remaining 90% has a substantially smaller moment of inertia.

If we substitute Equation (7) to the right side of Equation (5)

(10) $$\frac{P_{\mathrm{area},\mathrm{Gi}}-P_{\mathrm{area},\mathrm{Li}}}{I_{\mathrm{area},i}\;\omega_{G}}=\frac{P_{\mathrm{area},\mathrm{Gi}}-P_{\mathrm{area},\mathrm{Li}}}{\frac{\sum \left(2\;H_{\mathrm{Gi}}\;S_{\mathrm{Gi}}\right)}{{\omega_{G}}^{2}}+\frac{\sum \left(2\;H_{\mathrm{Li}}\;S_{\mathrm{Li}}\right)}{{\omega_{G}}^{2}}\;\omega_{G}}$$

After simplification, we obtain:

(11) $$\frac{\left(P_{\mathrm{area},\mathrm{Gi}}-P_{\mathrm{area},\mathrm{Li}}\right)\;\omega_{G}}{2\;\sum \left(H_{\mathrm{Gi}}\;S_{\mathrm{Gi}}\right)+\left(H_{\mathrm{Li}}\;S_{\mathrm{Li}}\right)}$$

Assuming that the power factor is one (S≈P):

(12) $$\frac{\left(P_{\mathrm{area},\mathrm{Gi}}-P_{\mathrm{area},\mathrm{Li}}\right)\;\omega_{G}}{2\;\sum \left(H_{\mathrm{Gi}}\;P_{\mathrm{Gi}}\right)+\left(H_{\mathrm{Li}}\;P_{\mathrm{Li}}\right)}=\frac{2\;\pi \;50\mathrm{Hz}}{2}\;\frac{\left(P_{\mathrm{area},\mathrm{Gi}}-P_{\mathrm{area},\mathrm{Li}}\right)}{\sum \left(H_{\mathrm{Gi}}\;P_{\mathrm{Gi}}\right)+\left(H_{\mathrm{Li}}\;P_{\mathrm{Li}}\right)}$$

which shows that this part of Equation (7) is affected by both generation and load mix.

With similar steps, the remaining elements of Equation (5) can be rewritten:

(13) $$\begin{array}{lll} {\ddot{\theta }}_{\mathrm{area},i}+\alpha \;{\dot{\theta }}_{\mathrm{area},i} & = & \frac{\omega_{G}}{2}\;\frac{\left(P_{\mathrm{area},\mathrm{Gi}}-P_{\mathrm{area},\mathrm{Li}}\right)}{\sum \left(H_{\mathrm{Gi}}\;P_{\mathrm{Gi}}\right)+\left(H_{\mathrm{Li}}\;P_{\mathrm{Li}}\right)}\\ & + & \frac{\omega_{G}}{2}\;\frac{K_{\mathrm{area},i}}{\sum \left(H_{\mathrm{Gi}}\;P_{\mathrm{Gi}}\right)+\left(H_{\mathrm{Li}}\;P_{\mathrm{Li}}\right)}\;\sum_{j=1}^{N}A_{\mathrm{ij}}\;\sin\left(\theta_{j}-\theta_{i}\right) \end{array}$$

Equation (13) is the form, which we used in the simulation code of Hungarian High Voltage (HU-HV) power-grid.

We have studied three different types of networks, by gradually increasing the heterogeneity:

- 2D square lattices, with periodic boundary conditions, simulating homogeneous electric power-grids using Equation (1).
- The 4941 node power-grid of the western states of the US (US-HV) [43] with Equation (1).
- A 418 node Hungarian HV electric power grid, deduced from the Hungarian Transmission System Operator (MAVIR) detailed database, using Equation (13).

We evaluated at each time step the actual power flow along the transmission lines and compared it to the available capacity of the edges of the network as in [38]. The flow of the power from edge *j* to *i* with the generalized coupling

(14) $$K_{ij}^{\prime }=\frac{\omega_{G}}{2}\;\frac{K_{\mathrm{area},i}A_{\mathrm{ij}}}{\sum \left(H_{\mathrm{Gi}}\;P_{\mathrm{Gi}}\right)+\left(H_{\mathrm{Li}}\;P_{\mathrm{Li}}\right)}$$

is described by

(15) $$F_{ij}=K_{ij}^{\prime }\sin\left(\theta_{j}-\theta_{i}\right)\;.$$

The overload condition is expressed by a comparison with a fraction T∈[0,1] of the maximum flow

(16) $$|F_{ij}|>TK_{ij}^{\prime }\;.$$

During the solution of the equation of motion, we checked this condition at each time step. In case the power flow of the line exceeded a pre-set threshold, we cut the line by resetting the adjacency matrix elements $A_{ij}=A_{ji}=0$. These thresholds can be selected by the settings of transmission line protection, which are responsible for tripping the line in case of instantaneous overloads.

We applied fourth order Runge–Kutta method (RK4 from Numerical Recipes) [44] to solve Equation (13) on various networks. Step sizes: Δ=0.1,0.01,0.001,0.0001 and the convergence criterion $\epsilon =10^{-12}$ were used in the RK4 algorithm. Generally, the Δ=0.001 precision did not improve the stability of the solutions, except at large *K*-s, while Δ=0.1 was insufficient, so most of the results presented here are obtained using Δ=0.01. In case of the 2D and US-HV grids, we applied $\langle \omega_{i}^{0}\rangle =0$ self-frequencies. Due to the Galilean invariance of Equation (1) we can gauge out the mean value in a rotating frame.), while in the case of the HU-HV, the mean-values come from the first term of right hand side of Equation (13). For modeling uncorrelated fluctuations, we added random numbers $\xi_{i}$ to the self-frequencies $\langle \omega_{i}^{0}\rangle$, following unit variance Gaussian distribution. In order to model correlated fluctuations, we added $\xi_{i}$-s with exponential tail distributions of the form: $p\left(\xi_{i}\right)=\left|\kappa \exp\left(-\xi_{i}\right)\right|$.

The initial state was fully synchronized: $\theta_{i}\left(t\right)=0$, $\dot{\theta_{i}}\left(t\right)=0$, but for testing the hysteresis we used uniform random distribution of phases: $\theta_{i}\left(t\right)\in \left(0,2\pi \right)$. Note that these conditions do not correspond to a fixed point, which is characterized by the sum over all flows $\sum_{j}F_{ij}$ being equal to the generated power at each node *i*. Thermalization was performed by running the code for $10^{5}$ iterations. Following that, we perturbed the system by removing a randomly selected node in order to simulate a power failure event. After this, initial node removal the dynamics was simulated according to Equation (1) or Equation (2) and lines are cut dynamically, according to the criterion (16). We also tried such perturbations by line cuts, but these caused too small cascades for making statistical analysis. We also tried multiple, simultaneous random node removals, which caused larger, but identical, blackout distributions as the single node case. During the cascade simulations, which had the length of $t_{max}=10^{4}$ (Throughout the simulations we assumed dimensionless units for the time, but, in the case of the HU-HV, we had parameters, with real SI units; thus, here, time can be interpreted with units of *s*.) we measured the Kuramoto order parameter:

(17) $$z\left(t_{k}\right)=r\left(t_{k}\right)\exp i\theta (t_{k})=1/N\sum_{j}\exp[i\theta_{j}\left(t_{k}\right)]\;,$$

by increasing the sampling time steps exponentially:

(18) $$t_{k}=1+1.08^{k}\;,$$

where $0\leq r(t_{k})\leq 1$ gauges the overall coherence and $\theta (t_{k})$ is the average phase. We solved (1) numerically for $10^{4}-10^{6}$ independent initial conditions, with different $\omega_{i}^{0}$-s and determined the sample average: $R\left(t_{k}\right)=\langle r\left(t_{k}\right)\rangle$. We also recorded the total number of line failures $N_{f}$ of each sample and calculated the probability distribution $p(N_{f})$ of them. In the steady state, which we determined by visual inspection of the mean values, we measured the standard deviation: $\sigma_{R}$ of $R(t_{k})$ in order to locate the transition point.

### Description and Analysis of the Power-Grids

**Figure 2:**
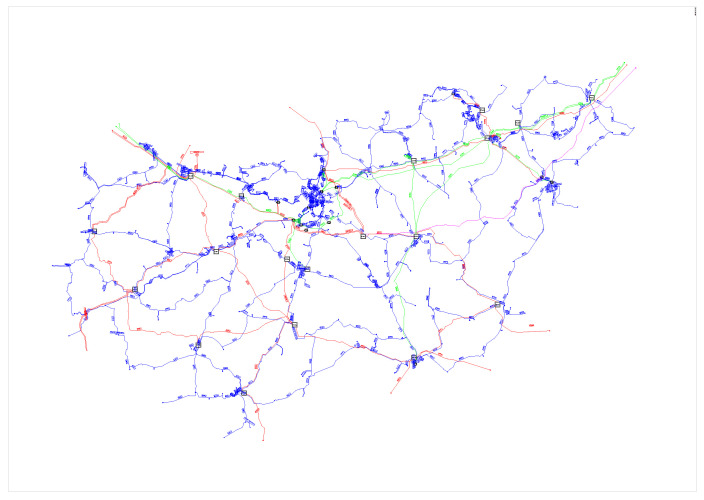
The topography of Hungarian transmission (750 kV—purple, 400 kV—red, 220 kV—green) and sub-transmission (120 kV—blue) networks.

**Figure 3:**
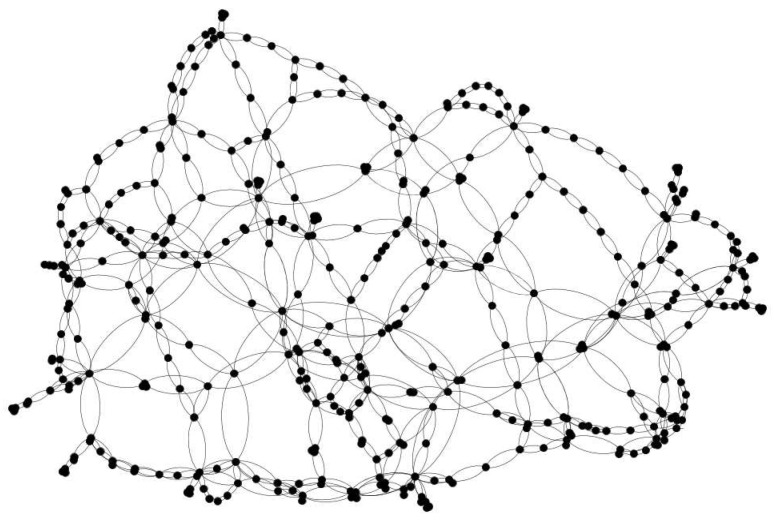
The topology of the HU-HV grid. Note, that in transmission networks unidirectional lines may occur, but here double lines were also modeled as single connections.

The authors have relied dominantly on the data provided by MAVIR (see Figure 2) to create the model of the Hungarian HV power grid. Complete topology of 750, 400, and 220 transmission and 120 kV sub-transmission networks has been replicated with 418 nodes. The topology of these systems (see Figure 3) is mostly looped and meshed, with only a number of direct lines. The model includes approx. 50 larger power plants, 200 composite distributed generators, which represent units of mixed fuel (gas engines, solar photovoltaics, and wind turbines), and 200 loads. The generation mix, the share of converter-based generation units in the portfolio, and the value of $K_{i}$ couplings were determined using statistics of the Hungarian Energy and Public Utility Regulatory Authority and MAVIR, while $P_{i}$ and $I_{i}$ values were set according to empirical distributions created from historical data. $H_{\mathrm{Gi}}$ and $H_{\mathrm{Li}}$ were 5.5 [s] and 0.36 [s], respectively.

We determined some basic topology characteristics [45] of this graph while using the Gephi tool [46]. The N=418 nodes of the network are interconnected via E=1077 undirected links. The average degree is: 〈k〉=2.595 and the exponent of the cumulative degree distribution is: γ=1.51(4), which renders this network just at the threshold of robust/fragile: γ=3/2, according to the definition by [11]. Note that, in the publication [10], only the 220 and 400 kV infrastructure of the Hungarian HV network was considered, which is a smaller sub-network with N=40, possessing more fragile geometry than the model used for present paper.

The HU-HV is a highly modular network with modularity quotient Q=0.8, being defined by

(19) $$Q=\frac{1}{N\langle k\rangle }\sum_{ij}\left(A_{ij}-\frac{k_{i}k_{j}}{N\langle k\rangle }\right)\delta \left(g_{i},g_{j}\right),$$

where $A_{ij}$ is the adjacency matrix and δ(i,j) is the Kronecker delta function. The Watts–Strogatz clustering coefficient [47] of the network of *N* nodes is

(20) $$C=\frac{1}{N}\sum_{i}2n_{i}/k_{i}\left(k_{i}-1\right)\;,$$

where $n_{i}$ denotes the number of direct edges interconnecting the $k_{i}$ nearest neighbors of node *i*, C=0.076 is about 10 times higher than that of a random network of same size $C_{r}=0.0062$, defined by $C_{r}=\langle k\rangle /N$. The average shortest path length is

(21) $$L=\frac{1}{N(N-1)}\sum_{j\neq i}d\left(i,j\right)\;,$$

where d(i,j) is the graph distance between vertices *i* and *j*. In case of HU-HV, this is L=8.163, which is somewhat larger than that of the random network of same size: $L_{r}=6.2244$ obtained by the formula [48]

(22) $$L_{r}=\frac{\ln(N)-0.5772}{\ln\langle k\rangle }+1/2\;.$$

Accordingly, this is a small-world network, according to the definition of the coefficient [49]:

(23) $$\sigma =\frac{C/C_{r}}{L/L_{r}}\;,$$

because σ=9.334 is much larger than unity.

We have also studied the dynamical behavior on the western states power-grid of US-HV that we downloaded from [43]. This is a standard modular network, in which all of the transmission lines are bidirectional and identical, but other (distribution...etc.) lines are omitted. Nodes are also identical and featureless. The network invariants are summarized in the Table 3.

As we can see, this network is about 10 times larger than the HU-HV, but it exhibits similar network invariant values. The small world coefficient is large again: σ=18.88. The cumulative degree distribution is: γ=1.246, categorizing it a robust network, by static topological sense. Later, we shall investigate if this holds in the dynamical sense, in the presence of fluctuating energy resources.

**Figure 4:**
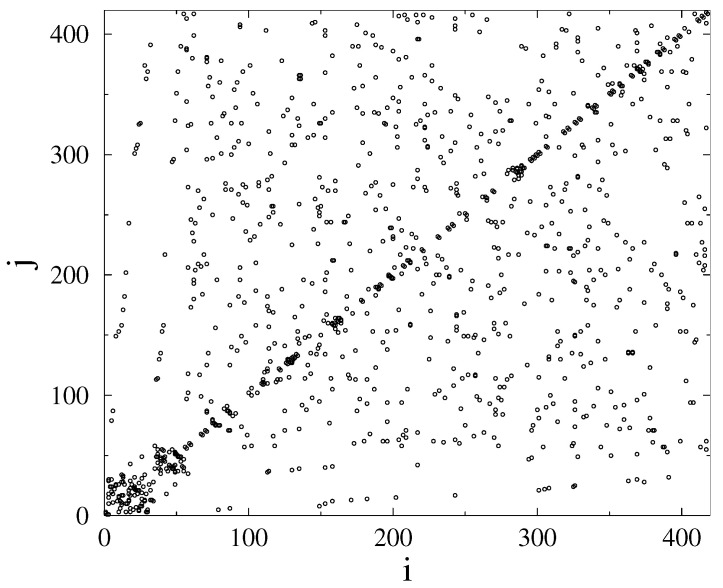
Adjacency matrix of of the HU-HV grid. Circles mark nodes *i* and *j* connected.

**Figure 5:**
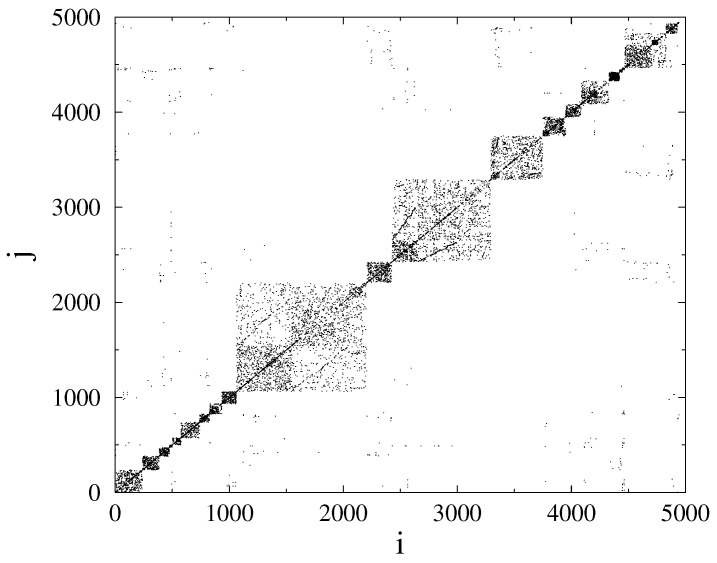
Adjacency matrix of of the US-HV grid. Dots mark nodes *i* and *j* connected.

By looking at the adjacency matrix of the N=418 node HU-HV grid (Figure 4), we can see some blocks, especially for node numbers i≤40, corresponding to the sub-network, considered in [10], but, many other connections, resembling like a random structure, are also present. This is in contrast with the US-HV grid (Figure 5), where a more regular, HMN structure is visible. This does not mean the lack of HMN structure of the Hungarian system had we considered lower levels [3], but suggests a more random-like structure. Note that, in ref. [10], more random-like structures were found to be more robust.

## 3. Simulation Results

In this section, we will compare the results of the threshold Kuramoto simulations on different networks, by gradually increasing the spatial heterogeneity. We start from the homogeneous two-dimensional square lattice, in which the self-frequencies at different nodes only vary randomly. Subsequently, we move on to the standard US-HV power grid, also possessing topological heterogeneity. Finally, we consider the most realistic HU-HV, in which even the edges and node parameters change. We also supplement the HU-HV case with a feedback control study.

### 3.1. The Two-Dimensional Square Lattice

**Figure 6:**
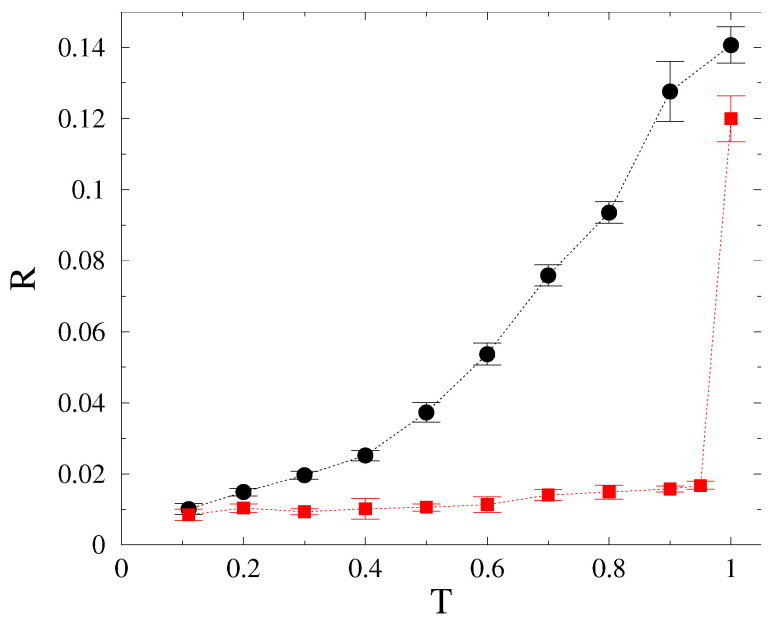
Hysteresis of the Kuramoto order parameter on the 2D square lattice as the function of threshold at K=10. Upper branch bullets corresponds to synchronized initial state, while lower branch boxes to random initialization of $\theta_{i}$.

We have run the analysis using Equation (1) on $N=10^{4}$ and $N=4\times 10^{4}$ sized lattices, with periodic boundary conditions, in order to determine the consequences of topological heterogeneity. Here, we found signatures of first order synchronization transitions with wide hysteresis loops (see Figure 6). This is similar to the results that we obtained for the 2D second order Kuramoto in Ref. [3] without allowing line failures. The hysteresis means a difficulty of the restoration of the synchronous state following a blackout collapse.

**Figure 7:**
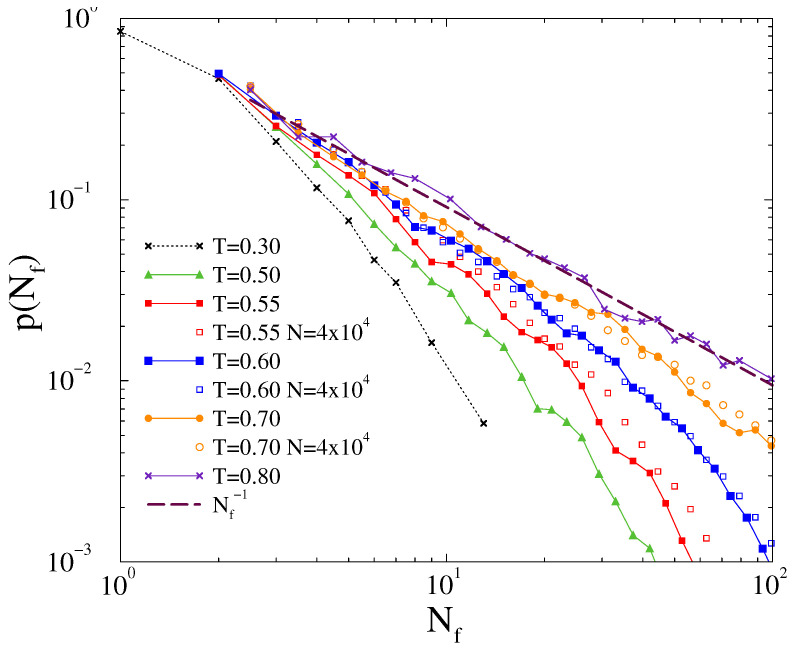
Probability distributions of line failures for different failure thresholds (*T*), shown by the legends, at K=10. Closed symbols: $N=10^{4}$, open symbols: $N=4\times 10^{4}$. The dashed line shows a PL fit for the T=0.8 data, corresponding to the singular $p\left(N_{f}\right)≃1/N_{f}$ case, lying in the disordered phase.

Synchronization transition is only visible clearly at lower global coupling values. For K>10, the transition becomes smooth, the system remains mostly in the partially synchronized state. There are no signatures of PL-s in the Kuramoto order parameter R(t) curves and they converge quickly to their steady state values for all *K* values. The distribution of the total number of line failures also do not exhibit PL-s, but break down exponentially, or follow the singular $p\left(N_{f}\right)≃1/N_{f}$ behavior, corresponding to the synchronization state for $K>K_{c}≃0.7$ before the finite size cutoff (see Figure 7).

By increasing the system size from $N=10^{4}$ to $N=4\times 10^{4}$, the results did not change, as shown in the figure for the K=0.55,0.6,0.7 coupling cases. Note that the average size of the blackouts decrease with *T*, because several links are already removed during the thermalization process before the actual cascade simulations started.

### 3.2. The US-HV Power-Grid

**Figure 8:**
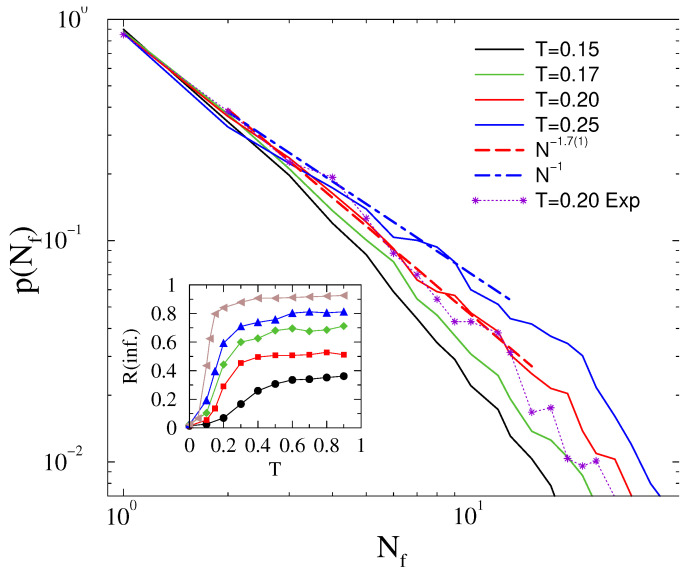
Probability distribution of line failures for different thresholds at K=30 shown in the legends in case of the US power-grid. Lines corresponds to Gaussian distributed $\omega_{i}^{0}$-s, while star symbols to the exponentially distributed self-frequencies in the case of T=0.2. Dashed lines show power-law fits for the scaling region, being determined by visual inspection. The inset shows R(t→∞) as the function of time, for K=10,20,30,40,70 (bottom to top curves).

**Figure 9:**
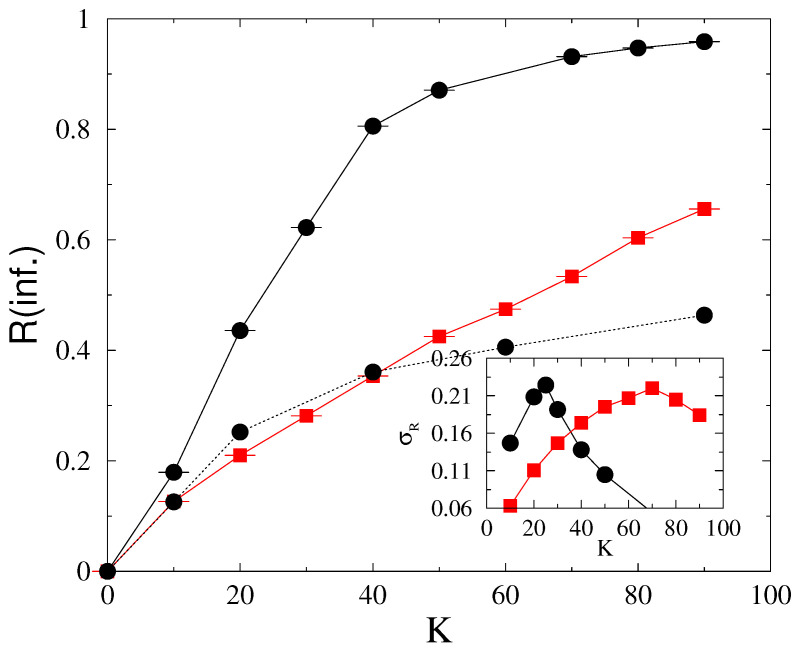
Steady state order parameter as the function of *K* for T=0.3 in case of the US-HV. Black bullets are for Gaussian, while red boxes are for exponential tailed $g(\omega_{i}^{0})$ self-frequency distributions. The two branches of Gaussian correspond to ordered and disordered initial states representing a hysteresis loop, closing at K>400. The inset shows the fluctuations, $\sigma_{R}$ of the same.

Next, we performed dynamical simulations using Equation (1) on the US-HV power grid, which also has topological heterogeneity, but the lines and nodes are identical. We found smooth crossover from desynchronization to partial synchronization by increasing the global coupling *K*, as in case of 2D and the US-HV without line failures [3]. On the other hand, there is a sudden jump by increasing the threshold from T=0 to small values. The inset of Figure 8 summarizes the steady state values for various *K*-s as the function of threshold *T*. We can find a transition region for T<0.5, which we shall investigate in more detail. In Figure 9, we show the steady state behavior at fixed T=0.3 as the function of *K*. At this threshold, the fluctuation peak $\sigma_{R}$ marks a transition point at K≃25. One can also see the lower part of a hysteresis loop, closing at K>400, corresponding to synchronous and asynchronous initial conditions. In case of exponential tailed $g(\omega_{i}^{0})$, the Kuramoto order parameter decreases and the transition point shifts to larger coupling K≃70.

We have also investigated the dynamical behavior at K=30, near the transition point. As Figure 8 shows for Gaussian $p(\omega_{i}^{0})$-s we find PL tailed $p(N_{f})$ line failure distribution at T=0.2, which can be fitted by $N_{f}^{-1.7(1)}$, in agreement with the empirical data and simulations by Ref. [6,7]. However, this PL breaks down rather early, for $N_{f}<30$, due to the finite size of the network. Another PL: $N_{f}^{-1}$ can be fitted for the T=0.25 curve, but this corresponds to a singular distribution, corresponding to the disordered phase, where any kind of large cascade might occur, being restricted by the finite grid size.

By changing the Gaussian $p(\omega_{i}^{0})$-s to an exponential tailed one we cannot see difference in the line failure distribution at T=0.2, as shown in Figure 8. Of course, in the steady state the synchronization drops substantially, as demonstrated in Figure 9. Note that a more realistic US-HV power-grid, containing node and line heterogeneity data would be needed to make a comparison with real life. In the lack of this, we now turn towards the Hungarian HV power-grid, for which we could access these data, although for a smaller network now. Still, a comparison, in which we gradually increase the heterogeneity from 2D across US to HU power grids provides a useful insight into the effects of heterogeneity on the synchronization behavior of these models.

### 3.3. The Hungarian HV Power-Grid

Next, we studied Equation (13) on the empirical HU-HV power-grid, deduced from the Hungarian database of MAVIR. At first, inertia constants of nodes with purely load connections were set to $H_{i}=0.36$ s, but very low-level synchronization was obtained, even for T=1. This is the consequence of high-level heterogeneity destroying the synchronization. Thus, we modified the model by equalizing inertia constants as $H_{i}=5.5$ s for most of the nodes. The exceptions were nodes with purely generation connections, where inertia constants were selected based on Table 1 and cross-border connections, where inertia constants reflect a different composition of generation portfolio in neighboring countries (ranging from 2.25 s to 4.5 s).

**Figure 10:**
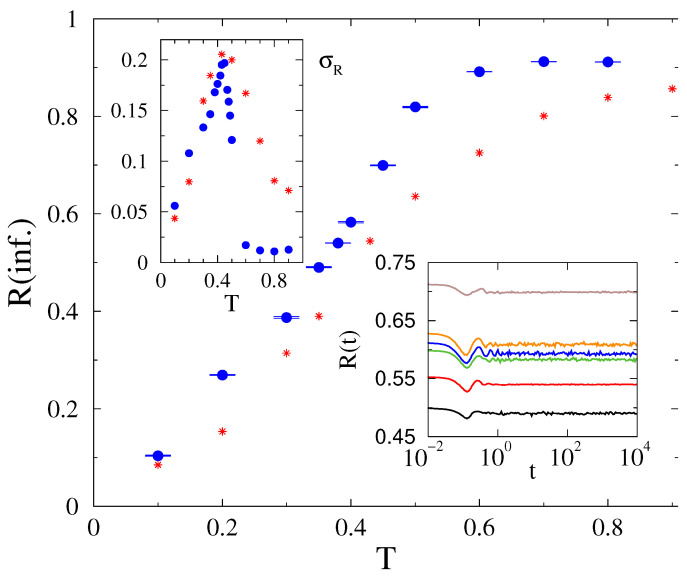
Kuramoto order parameter in the HU-HV power-grid as the function of threshold, bullets: Gaussian $g(\omega_{i}^{0})$, stars: exponential tailed fluctuations. The upper inset shows $\sigma_{R}$ of the same. The lower inset shows the time dependence in case of $g(\omega_{i}^{0})$ at T=0.35,0.38,0.40,0.42,0.43,0.45 (bottom to top curves).

Now we could find reasonable average order parameters and a synchronization transition, as shown in Figure 10. The peak of the standard deviations of the order parameter marks a transition point at $T_{c}=0.44\left(1\right)$. If we replace $g(\omega_{i}^{0})$ from Gaussian to exponential tailed self-frequencies, the order parameter decreases and $\sigma_{R}$ increases, but the peak does not move a lot.

**Figure 11:**
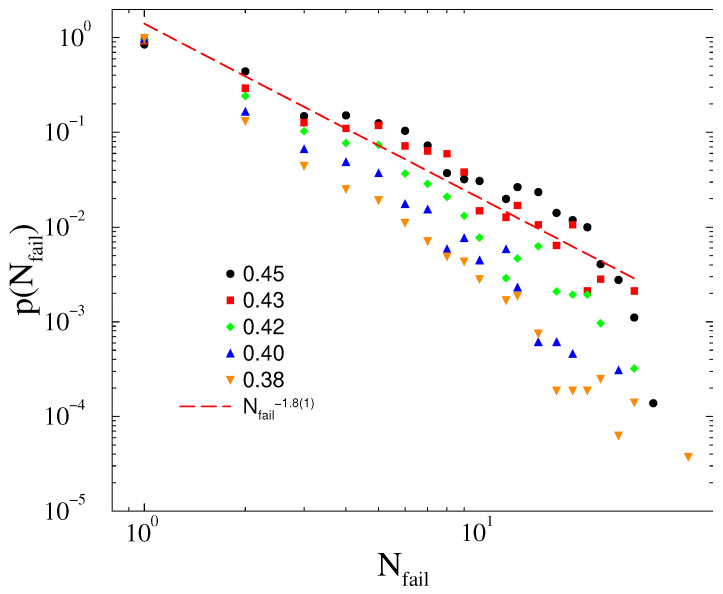
Probability distribution of line failures for different thresholds, as shown in the legends in case of the HU-HV power-grid. The dashed line shows a power-law fit for scaling region of the T=0.43 results.

The probability distribution of line failures exhibit PL behavior tails at $T_{c}=0.43$, which are characterized by the exponent $\tau_{N}\geq 1.8\left(1\right)$, close to the blackout failure exponent, as shown on Figure 11. Below, the transition is hard to determine if other PL-s with cutoffs or a simple exponential decay happens given the small system sizes. We favor the former scenario, but plan to test it in the future, when larger power-grids and more computation resources will be at our disposal. Note that the load dependent PL exponents have been also advanced in the case of DC threshold models of power grids [50]. Later, we will investigate whether a feedback mechanism can stabilize the synchronization of the model with fully heterogeneous inertia. Such feedback is present in real system, so it is an important issue to investigate.

**Figure 12:**
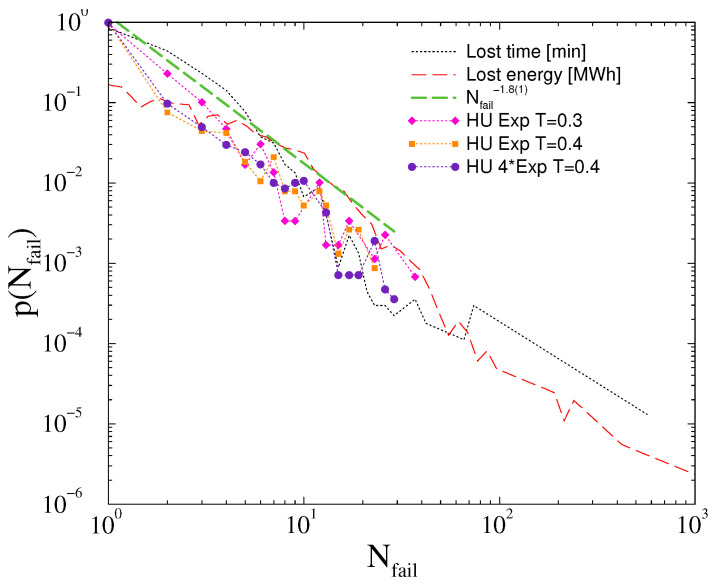
The same as in Figure 11, in the case of exponential tailed self-frequency fluctuations. The green dashed line shows a power-law fit for the scaling region of the T=0.4 threshold result shifted up for better visibility. For comparison, we also show empirical distributions for the lost time (black dots) and lost energy (orange dashed line) obtained from the MAVIR database.

The line failure distributions of the HU-HV power-grid seems to be quite insensitive for replacing the Gaussian self-frequency fluctuations to exponential ones. As Figure 12 shows, the $p(N_{f})$ distributions decay with the same PL tails as before, being characterized by the exponent $\tau_{N}\geq 1.8\left(1\right)$, even up to κ=4 amplitudes. Therefore, the HU-HV power-grid model seems to be robust against large fluctuations.

For completeness, we also show a comparison of our model calculations with the lost time (min) and rescaled lost power (MW), obtained from planned and unplanned outages of the Hungarian HV networks. The metric, described by the curve “lost energy” is also known as energy not served (ENS), a widely accepted fundamental index of power system reliability. ENS is defined as the expected amount of energy not being served to consumers by the system during the period considered due to system capacity shortages or unexpected severe power outages. Statistics of the Hungarian transmission system were used to determine the probability distribution of this metric. Using the same dataset, for each outage event, we determined the amount of time that was necessary for restoring operation: this is shown by the curve “lost time”. Following appropriate rescaling, we can see remarkable agreement of the probability distributions with those obtained by our simulations.

### 3.4. Instantaneous Feedback Control On the HU-HV Power-Grid

**Figure 13:**
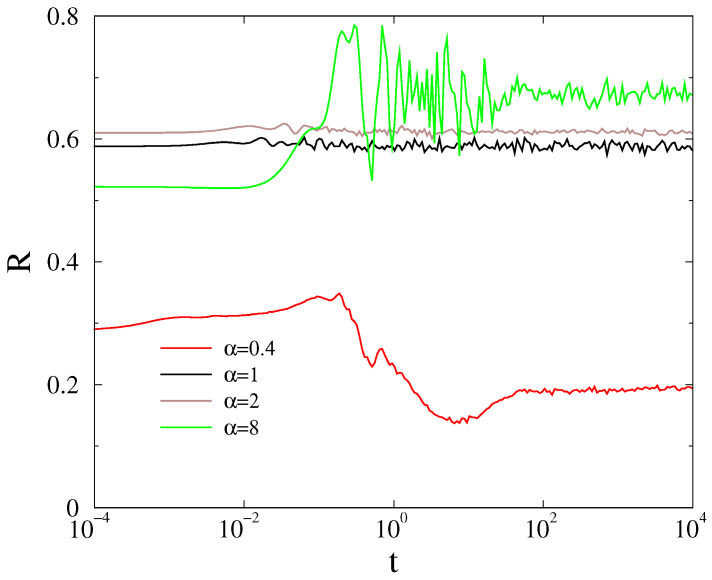
Effect of the instantaneous feedback by increasing α=0.4,1,2,8 in the HU-HV power-grid with heterogeneous inertia $H_{i}$ at T=0.43.

As we mentioned, the application of Equation (13) on the HU-HV power-grid with real inertia constants shown in Table 1 and Table 2 leads to low synchronization levels, the strong heterogeneity prevents to achieve realistic synchronization values. In the recent study by [24], the effects of different feedback control mechanisms have been compared. It was shown that time delayed feedback provides efficient ways to improve synchronization, but an instantaneous feedback can also make the system more stable. Without going into the details of such analysis, which is out of the scope of our present interest, we just show how an instantaneous feedback alters our results. This can done be rather easily, since the equation of motion is almost like the original one: Equation (1):

(24) $$\dot{\omega_{i}}\left(t\right)=\omega_{i}-\alpha \dot{\theta_{i}}\left(t\right)+K\sum_{j=1}^{N}A_{ij}\sin\left[\theta_{j}\left(t\right)-\theta_{i}\left(t\right)\right]-g\alpha \dot{\theta_{i}}\left(t\right)\;$$

with the addition of a new term, describing the feedback with gain value *g*. This can be fused with the dissipation term $\alpha \dot{\theta_{i}}\left(t\right)$, thus modeling a simple instantaneous feedback means enhancement of α in our simulations. Figure 13 shows the time dependence of the order parameter by increasing α in the case of the HU-HV power-grid model using real, heterogeneous $H_{i}$ values from Table 1 and Table 2. As we can see, this feedback mechanism increases R(t), but the precise solution requires much smaller step sizes due to the high amplitudes of the derivatives by the integration steps. In Figure 13, we showed R(t) results using δ=0.0001 precision, averaged over 500 samples, because even δ=0.001 proved to be insufficient. Unfortunately, generating $p(N_{f})$ distributions with this precision is very slow and a better, time delayed or targeted mechanism would be needed to see possible scaling of cascade sizes.

### 3.5. Summary of Simulations

In this section, we have shown the results of extended dynamical simulations of the threshold synchronization, modeling power-grids on different topologies. We have found numerical evidences that line failure distributions can exhibit PL tails, in agreement with real statistics, by applying the second order threshold Kuramoto model on heterogeneous networks. Although the second order Kuramoto model itself exhibits a discontinuous transition from chaotic to partially synchronized state, by increasing the oscillator couplings the desynchronization cascade size distributions of the threshold version show dynamical critical like behavior at and below the transition, similar to what was obtained by SOC DC models earlier. This can happen if the heterogeneity in the system is moderately strong. For low heterogeneity, as in case of the square lattice, we have not found signatures of scale-free tails. For too strong heterogeneity, as in case of the HU-HV model with real inertia, the level of synchronization remained very low. This could be compensated by equalizing the inertia terms or by a feedback mechanism. We have also shown that the application of exponentially distributed self-frequencies do not alter the exponents of PL tails, but, of course, decrease the synchronization order parameter. Thus, they pose a moderate risk on the stability of power-grids.

## 4. Conclusions

Power-grids are becoming increasingly heterogeneous, as renewable (solar, wind, ... etc.) small suppliers are connected. Therefore, the danger of failures that are caused by desynchronization is of a great concern. Failure data of large power-grids have shown blackout size distributions with power-law (PL) tails. Previous simulations could explain this using power threshold cascade models, assuming self-organized criticality. In these DC models, the power redistribution, following a line or node cut, is described by a fixed amount of load. We have studied the stability of phase and frequency synchronized steady-states of realistic, Hungarian, and US high voltage power grids while using dynamical simulations of the swing-equations, which describe the real power redistribution in AC electric networks. Earlier, we have shown that heterogeneity can generate power-law desynchronization duration distributions without the assumption of criticality [3].

Now, we obtained roughly universal PL failure tails, without fine tuning to a critical point: i.e., at different thresholds (T), global couplings (K), and self-frequency distributions, for the 4941 node US and the 418 node HU-HV networks. The fitted exponents agree with those of the HU failure time data and other world-wide measurements. While the synchronization values dropped, the US and HU grid cascade size distributions both seem to be insensitive to such stronger fluctuations.

We emphasize, that we do not rule out a SOC mechanism, which tunes the network into the neighborhood of the synchronization transition point, as the consequence of power supply/demand competition, but show that this parameter region is extended, due to heterogeneity and load dependent PL exponents potentially arising. The lack of PL-s in the case of the homogeneous 2D square lattice shows that heterogeneity must be taken into account, simple homogeneous models cannot describe scale-free behavior of outages.

We also found that too strong heterogeneity of inertia destabilizes the power-grid and reliable synchronization cannot be sustained without feedback. Applying simple zero lag feedback were insufficient in our model; possibly, a time-delayed feedback control would be necessary, as suggested in [24], which should be the target of further research. This feedback is supposed to represent the frequency response of generators and loads. In the case of generators, units providing primary reserve (or Frequency Containment Reserve) provide a practically immediate response based on the steepness (MW/Hz) of their open-loop control characteristic. Similarly, the behavior of loads during frequency disturbances can be described by their respective correlation factor (MW/Hz); however, their response is usually slightly delayed. Still, without the this feedback, our model is capable to describe short time scales, which can be interesting for high variability systems with rapid changes, coming from large fluctuations of renewable resources.

Our future work will focus on further extensions of the presented model. To describe the stabilization of synchronization, a retarded model will be implemented, which has larger inertial feedback, thus compensating for the decrease of inertia due to renewable generation. Using the methods of complex network and hybrid tools, a better insight can be gained into robustness and vulnerability issues of power systems. Such multi-level network analysis has proven to be useful previously, as coupling level of different networked infrastructures may increase and decrease stability, depending on the actual level. Literature is yet to provide a validation of European power system failures in the presence of large share of distributed generation. It was also shown that tools, specifically designed for power system analysis, outperform the methods that are solely built on topological connections. This gap between the two approaches is to be examined in detail by the authors, while taking into consideration realistic network topologies and power flows, extreme failure statistics and the theory of self-critical systems.

The data-sets generated during and/or analyzed during the current study are available from the corresponding author on reasonable request.

## Figures and Tables

**Table 1:** Typical inertia constant of power plant types.

Production Type	*H* [s]
Nuclear	6
Combined cycle gas turbine	5.5
Single-shaft gas turbine	4.5
Large-scale hydro	3
Diesel genset	2
Converter-based units	0

**Table 2:** Typical inertia constant of certain consumers [41].

System	*H* [s]
Direct-on-line induction motor and compressor	1
Direct-on-line induction motor and conveyor belt	0.6
Direct-on-line synchronous motor and compressor	1
Variable speed drive	0
Lighting	0

**Table 3:** Network invariants of the US-HV grid.

*N*	*E*	*L*	〈k〉	$L_{r}$	*C*	$C_{r}$
4194	6594	2.67	18.7	3.15	0.08	0.005
